# Delayed surgery and health related quality of life in patients with proximal femoral fracture

**DOI:** 10.1038/s41598-023-33592-3

**Published:** 2023-07-10

**Authors:** Angela María Merchán-Galvis, David Andrés Muñoz-García, Felipe Solano, Julián Camilo Velásquez, Nelson Fernando Sotelo, David Alejandro Molina, Juan Pablo Caicedo, Juan Manuel Concha, José Andrés Calvache, María José Martínez-Zapata

**Affiliations:** 1 Iberoamerican Cochrane Centre-Public Health and Clinical Epidemiology, IIBSant Pau, Barcelona, Spain; 2 Department of Social Medicine and Family Health, Universidad del Cauca, Popayán, Colombia; 3 Department of Anaesthesiology, Universidad del Cauca, Popayán, Colombia; 4 Hospital Universitario San José, Popayán, Colombia; 5 Department of Surgical Sciences, Universidad del Cauca, Popayán, Colombia; 6 Department of Anesthesiology, Erasmus University Medical Center, Rotterdam, The Netherlands; 7 Centro de Investigación Biomédica en Red de Epidemiología y Salud Pública (CIBER of Epidemiology and Public Health), Madrid, Spain

**Keywords:** Health care, Medical research

## Abstract

This study aimed to establish factors associated with delayed surgery in patients with proximal femoral fracture and to assess patients’ health-related quality of life (HRQoL) after surgery including all-cause 6-months mortality. This was a single-center, observational, prospective cohort study that included patients with a proximal femur fracture. We described patients’ HRQoL measured by EuroQoL (EQ-5D-5L and EQ-VAS) questionnaire and perioperative complications (including mortality) 6 months after surgery. We included 163 patients with a mean age of 80.5 years, the majority were women and 76.1% reported falling from their own height. The mean time between hospital admission and surgery was 8.3 days (SD 4.9 days) and the mean hospital stay was 13.5 days (SD 10.4 days). After adjustment, the principal factor associated with delayed surgery was adjournment in surgery authorization (3.7 days). EQ-5D-5L index values and the VAS score at 1 month after surgery were 0.489 and 61.1, at 3 months were 0.613 and 65.8, and at 6 months 0.662 and 66.7 respectively. Mortality at 6 months of follow-up was 11% (18 patients). In conclusion, administrative authorization was the strongest associated factor with delayed time from hospital admission to surgery. HRQoL of patients with a proximal femoral fracture improved 6 months after surgery.

*Trial registration:* NCT04217642.

## Introduction

Proximal femoral fracture (PFF) is an important cause of morbidity, disability, and mortality in the elderly. Furthermore, it is a disease with high costs for the health system^1,2^. Those who suffer hip fracture usually have significant functional deterioration and approximately 8% of them reach prostration, especially when the surgical procedure is delayed^3^. Besides, PFF also affects mental health which is an important determinant of mortality risk^4,5^. Loss of independence by reducing mobility and functional capacity persists months after the diagnosis, with a significant decrease in health-related quality of life (HRQoL)^6–9^.

Given the demographic transition that Colombian population is experiencing, including a progressive increase in the elderly and a high prevalence of chronic non-communicable diseases, an increase in the incidence of PFF is expected^10^.

Time elapsed between the diagnosis of the hip fracture and its treatment considerably affects the survival of these patients. It is known that every two days of surgical waiting can double the probability of dying from complications such as pulmonary thromboembolism, pneumonia, urinary tract infections, cardiovascular complications, pressure ulcers, rejections of the osteosynthesis material, and over aggregated infection at the surgical site^11^. There is consensus that the time to surgery in the first 24–72 h after fracture decreases mortality and complications^12–14^. Some studies show that hip osteosynthesis performed more than 24 h after the fracture is associated with a higher risk of complications and costs, especially in intertrochanteric fractures with fixation^14,15^.

In developing countries, it is difficult to implement the surgery in a short time. There are few Colombian studies reporting time from hospital admission to surgery in hip osteosynthesis. Two retrospective studies carried out in Bogotá found a mean time to surgery from 5^16^ to 9^17^ days. For patients undergoing surgery in the first 48 h, they found a decrease in hospital stay and a decrease in mortality at 6 months^16,17^. However, the causes of surgery delay were not studied in them. Two reviews have described various patient (medical and socioeconomic) and system (operating room availability) factors related to surgical delay and adverse outcomes in patients suffering from hip fracture^18,19^.

Therefore, we performed a prospective cohort study including patients with incident PFF, with the objective of identifying factors associated with a delay in time to surgery. In addition, to describe HRQL and all-cause mortality assessed during the first 6 months after surgery.

## Methods

The HIP fracture in Cauca COhort (HIPCCO) study is a single center, prospective observational cohort study conducted in the traumatology service of the Hospital Universitario San José (HUSJ) of Popayán, Colombia between January 2019 and June 2021. We recruited consecutively adult patients admitted with a primary diagnosis of proximal femoral fracture who underwent emergency or scheduled surgery for surgical reduction of the fracture. Patients were excluded if they were at the end of their life, they have a cognitive impairment, limitations to understanding HRQoL questionnaires, or refused to participate.

HUSJ is a public hospital in a Colombian province, in the city of Popayán, attending the urban population (> 300,000 inhabitants) and a large rural area, including several indigenous tribes. Colombia has a mandatory “universal” national social insurance system including two main insurance schemes, a contributory one financed by payroll contributions and a subsidized scheme for the poorest population by general taxation. Membership in the health system is done through health-promoting entities.

Outcomes data were obtained through personal interviews with patients or by telephone (after hospital discharge) and a detailed review of hospital medical records by using a case report form. We collected administrative information, socio-demographic patients’ characteristics, co-morbidities, current and regular treatments. As part of the outcomes under assessment: HRQoL variables (EuroQol-5D-5L), days from fracture to admission, days of hospitalization, time to surgery, functional ambulation capacity and medical and surgical complications (including mortality).

The Charlson Comorbidity Index (CCI) was used as a method to quantify the severity of chronic diseases and predicts 10 years mortality^20^. The American Society of Anesthesiologists (ASA) classification system was used to assess perioperative risk^21^.

Sources of delay time to surgery were classified into two large groups: medical and administrative reasons. Medical reasons were decompensated baseline comorbidities (which needed to stabilize) or that added together gave a high ICC. Medical events generated during admission that required urgent treatment including urinary infection, pneumonia, etc., evaluations with other medical-surgical specialties or the requirement of complementary test. Considered administrative reasons were availability of osteosynthesis material, authorization by the health-promoting entities to perform the surgery, or the scheduling of the surgical shift.

To ascertain the HRQoL status, participants were interviewed six times during follow-up: At hospital admission, at the surgery day, at hospital discharge, one month, third month and six months after hospital discharge. HRQoL was assessed using the EuroQol-5D-5L questionnaire. Essentially it consists of 2 parts: the EQ-5D descriptive system and the EQ visual analogue scale (EQ-VAS)^22^. The EuroQol-5D-5L is a descriptive system with five domains (mobility, self-care, regular activities, pain/discomfort, and anxiety/depression) divided into five levels of severity: no problems, some problems, moderate problems, extreme problems, or unable (labeled 1–5; where 1 indicates that there is no problem and 5 unable). Each heath status description can be expressed into a summary index score based on cultural and national differences. This index score ranges from -0.654 to 1, in which 0 represents death, 1 represents full health and < 0 represents a health state considered worse than death^22^. We used the published index values of the United States population for calculating the EuroQol-5D-5L index values of the patients of the study^23^. The EQ-VAS records the patient’s self-rated health on a 20-cm vertical visual analogue scale evaluating the overall health status from 0—the worst—to 100—the best.

All patients included in the study received standard clinical practice. Written informed consent was required to participate in the study. The study was performed in accordance with the guidelines of the Declaration of Helsinki and were approved by the Research Ethics Committee of the Hospital Universitario San José Popayán (Record Number 8 Dec 14th, 2018). The study protocol was registered at Clinicaltrials.gov (NCT04217642). To enhance the completeness of this report we followed the STrengthening the Reporting of OBservational studies in Epidemiology STROBE checklist for cohort studies^24^.

We calculated proportions for categorical variables and mean (with standard deviation) (SD) or median (with interquartile range (IQR)) for the distribution of continuous variables. For comparisons between variables a student t test or a chisquare test were used depending on the quantitative or categorical values. Considering the perspective of gender (e.g. the incidence of osteoporosis and the tolerance to pain is higher in women than men), we stratified the analysis by sex to identify differences in the time from hospital admission to surgery, quality of life and mortality.

For explaining reasons of delay from admission to surgery, we performed a bivariate analysis. We considered potential patients-dependent (medical reasons) factors of delay such as: sex, age, request of an additional preoperative test, request for additional medical specialties assessment, Charlson comorbidity index, medical events generated during admission and decompensated basal pathology. Also, we considered potential patients-independent (administrative reasons) factors of delay such as: influence of insurance regime, osteosynthesis material non-availability, delay in the authorization of surgery and operating room scheduling delay.

We included the significant and clinically relevant factors identified (p < 0.05), in a multiple linear regression model by forward stepwise selection evaluating the fit of the model with the coefficient of determination (R2) to determine the increase in surgical delay time for each variable. We calculated the coefficients and their 95% confidence intervals (95% CI).

Mortality was reported as a cumulative incidence proportion. To identify the effect of sex on the rate of occurrence of mortality, a Cox regression model was built with the variables delay time and sex, obtaining the Hazard values Ratio with 95% CI. Survival was calculated from the time of hospital admission to 6 months after surgery by sex. The survival differences were calculated by Kaplan–Meier method using the Log rank test.

For the repeated measures of the EQ-5D-5L and EQ-VAS scores, two-way analysis of variance (ANOVA) was performed. The factors were group (women, men) and the evolution of the EQ-5D-5L and EQ-VAS scores pre-surgery, just after surgery, 30 days, 3 months and 6 months post-surgery. The ANOVA were undertaken by the Generalized Linear Models (GLM) procedure.

For all comparisons, we considered a p-value less than 0.05 statistically significant. Software used for data analysis included R Statistics (V.4.2.0) and IBM-SPSS (V.25).

## Results

**Figure 1:**
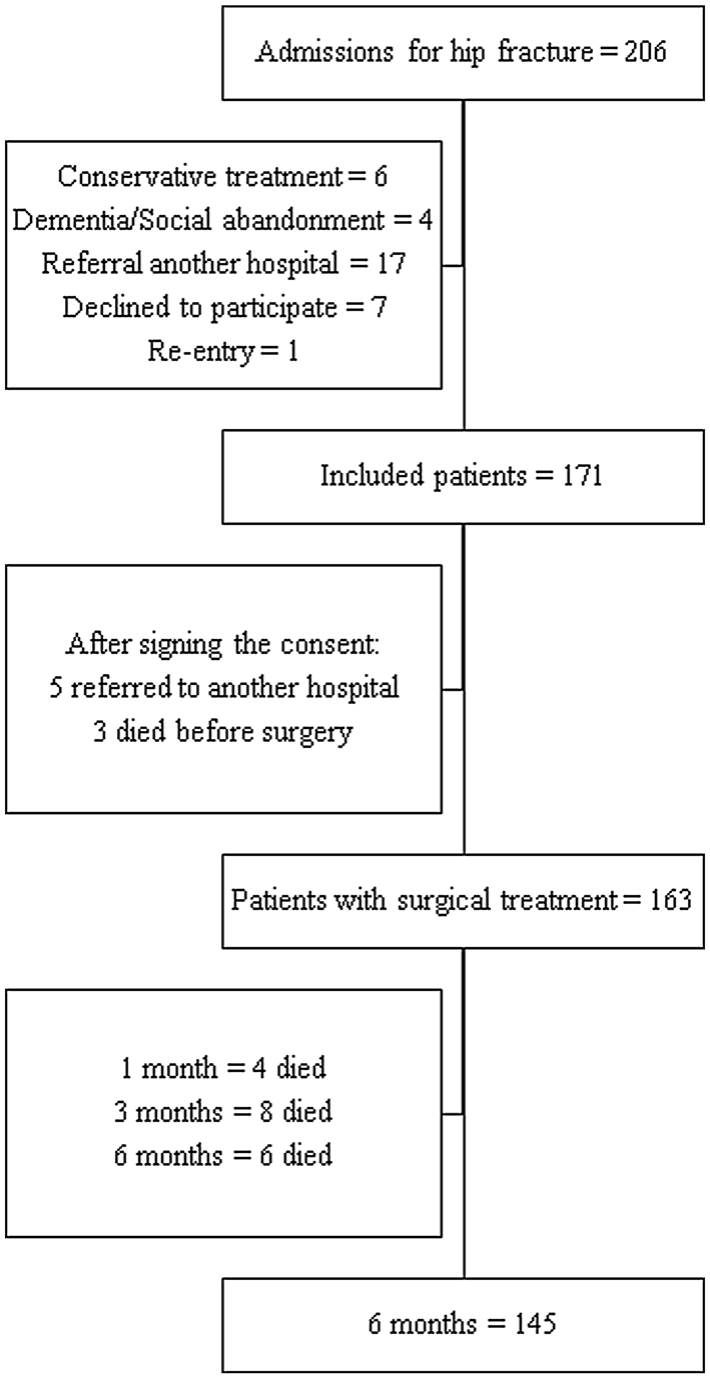
Selection process and mortality of patients with proximal femoral fracture diagnosis.

We screened two hundred and six patients admitted to the hospital for proximal femoral fracture during the 30 months of recruitment. Of these, seven patients (3.4%) refused to participate in the study, six (2.9%) received conservative treatment without surgery, three died before they could be operated on (3%) and 27 (13.1%) met one of the exclusion criteria. Finally, 163 patients were included (Fig. 1).

Mean age of the study participants was 78.9 years (SD ± 15.1 years); most patients were women (60.1%), Charlson’s Comorbidity Index at the time of surgery was 4.6 (SD ± 1.9) and 52.8% of patients were classified with stage II ASA (Table 1).

**Table 1:** Sociodemographic and clinical characteristics of patients at admission.

Variables	Total (n = 163)	Female (n = 98)	Male (n = 65)	p
Age (years)				
Median (IQR)	82 (75–88)	82.5 (76–88)	82.0 (70–88)	0.271
Mean (SD)	78.9 (15.1)	81.4 (9.1)	75.3 (20.7)	
Urban Origin, n (%)	105 (64.4)	65 (66.3)	40 (61.5)	0.532
Health care insurance regime, n (%)				
Subsidized	68 (41.7)	38 (38.8)	30 (46.2)	0.350
Contributive	95 (58.3)	60 (61.2)	35 (53.8)	
ASA, n (%)				
II	86 (52.8)	50 (51)	36 (55.4)	0.379
III	76 (46.6)	48 (49)	28 (43.1)	
IV	1 (0.6)	0 (0)	1 (1.5)	
BMI; Mean (SD)	24.7 (3.9)	25.5 (4.1)	23.5 (3.4)	0.025
Comorbidities, n (%)				
Cardiovascular	93 (57.1)	55 (56.1)	38 (58.5)	0.768
Endocrines	43 (26.4)	22 (22.4)	21 (32.3)	0.162
Neurologic	37 (22.7)	26 (26.5)	11 (16.9)	0.152
Musculoskeletal	30 (18.4)	13 (13.3)	17 (26.2)	0.038
Respiratory	18 (11.0)	8 (8.2)	10 (15.4)	0.150
Hematologic	4 (2.5)	0 (0)	4 (6.2)	0.013
None reported	32 (19.6)	20 (20.4)	12 (18.5)	0.759
Other	29 (17.8)	17 (17.3)	12 (18.5)	0.855
Charlson comorbidity index before surgery; Mean (SD)	4.6 (1.9)	4.4 (1.9)	4.9 (1.9)	0.316

*ASA* American Society of Anesthesiologists physical status, *BMI* Body Mass Index.

Most of the patients (96.9%) suffered a trochanteric fracture, compared to 3.1% who suffered a neck fracture, 76.1% reported falling from their own height and six patients suffered multiple traumatic injuries. Fracture reduction by intramedullary nail was the most common type of surgical procedure (63.8%). Most of the patients received spinal anesthesia (98%). A complication related to anesthesia was hypotension (24.5%) (Table 2).

**Table 2:** Clinical and surgical description of the fracture.

Variables	Total (n = 163) n (%)	Female (n = 98) n (%)	Male (n = 65) n (%)	p
Fracture mechanism				
Low energy trauma	124 (76.1)	84 (85.7)	40 (61.5)	0.004
High energy trauma	39 (23.9)	14 (14.3)	25 (38.5)	
Type of fracture				
Trochanter (extracapsular and extraarticular)	158 (96.9)	93 (94.9)	65 (100)	0.075
Neck (intracapsular and extra-articular)	5 (3.1)	5 (5.1)	0	
Type of surgical intervention				
Intramedullary nail or osteosynthesis	137 (84.1)	86 (87.8)	51 (78.5)	0.028
Partial hip replacement	16 (9.8)	10 (10.2)	6 (9.2)	
Total hip replacement	10 (6.1)	2 (2.0)	8 (12.3)	
Surgical complication				
Bleeding	11 (6.7)	6 (6.1)	5 (7.7)	0.696
Type of anesthesia				
General	3 (1.8)	2 (2.0)	1 (1.5)	0.008
Spinal	160 (98.2)	96 (98.0)	64 (98.5)	
Anesthesia complication				
Hypotension	40 (24.5)	16 (16.3)	24 (36.9)	0.003

We found significant differences related to a higher BMI in women (Table 1); men presented a higher number of high energy trauma, prosthetic replacements, hypotension related to anesthesia (Table 2), and mortality.

One hundred and twenty-four patients (76.1%) presented some postoperative adverse events and consequently, 45 of these patients had their hospital stay prolonged (36.3%). The most frequent complication was anemia (54%), followed by poor pain control (42.3%) and fluid and electrolyte disorder (20.9%). The total length of hospital stay was 13.5 days (10.4 days) (median 11.0 days, IQR = 7–15) and postoperative adverse events had a median duration of 2 days (IQR = 2–4) (Supplementary Table 1).

### Time from hospital admission to surgery

Mean time from hospital admission to surgery (delay time) was 8.3 days (4.9 days) (median 7 days, IQR = 5–10). In addition, mean time from fracture to hospital admission was 3.5 days (9.4 days) (median 1 day, IQR = 0.2–3). The total mean time between fracture and surgery was 11.8 days (10.5 days) (median 9 days, IQR = 6–14 days).

Administrative reasons were described as the main reasons to increase delay time and they were present in 159 patients (97.5%), including osteosynthesis material non-availability in 137 patients (84%), and operating room scheduling delay in 133 (81.6%). In addition, the request for an additional preoperative analysis (109 patients, 66.9%), and for an additional medical specialty consultation in 108 (66.3%) were found as the main patient-related medical reasons of delay. Patients with medical adverse events generated during admission compared to patients who did not present them had a longer delay time (10.8 vs. 7.5 days, mean difference − 3.3 days, 95% CI − 5.6 and 0.11, p = 0.051). After adjusting for covariates, the delay time was associated with the adjournment in the authorization of surgery and the medical events generated during admission (3.67 and 3.21 days more, respectively) (Table 3). The same causes and with the same relevance influenced hospital stay (Supplementary Table 2).

**Table 3:** Factors influencing delayed surgery.

	β	EE	p value	β Lower limit CI95%	β Upper limit CI95%
Bivariant model					
Age	− 0.003	0.026	0.908	− 0.053	0.048
Sex female	1.834	0.772	0.019	0.309	3.359
Subsidized insurance regime	1.181	0.775	0.129	− 0.348	2.711
Osteosynthesis material non-availability	0.885	1.048	0.400	− 1.185	2.955
Request of additional preoperative test	2.388	0.795	0.003	0.818	3.959
Charlson index	0.198	0.204	0.333	− 0.205	0.601
Operating room scheduling delay	0.019	0.993	0.985	− 1.941	1.979
Request of additional medical specialties assessment	2.429	0.791	0.002	0.868	3.991
Delay in authorization of surgery	3.271	0.881	0.000	1.531	5.012
Decompensated basal pathology	2.425	0.987	0.015	0.475	4.375
Medical events generated during admission	3.247	1.096	0.004	1.082	5.412
Final model*					
Delay in authorization of surgery	3.674	0.822	0.000	2.051	5.297
Medical events generated during admission	3.214	1.010	0.002	1.220	5.208
Request of additional medical specialties assessment	2.369	0.730	0.001	0.928	3.810
Decompensated basal pathology	2.037	0.897	0.025	0.265	3.809

### Mortality

Mortality at 1 month, 3 months, and 6 months of follow-up was 2.5%, 7.4%, and 11% respectively, being in men 16.9% and women 7.1%.

**Figure 2:**
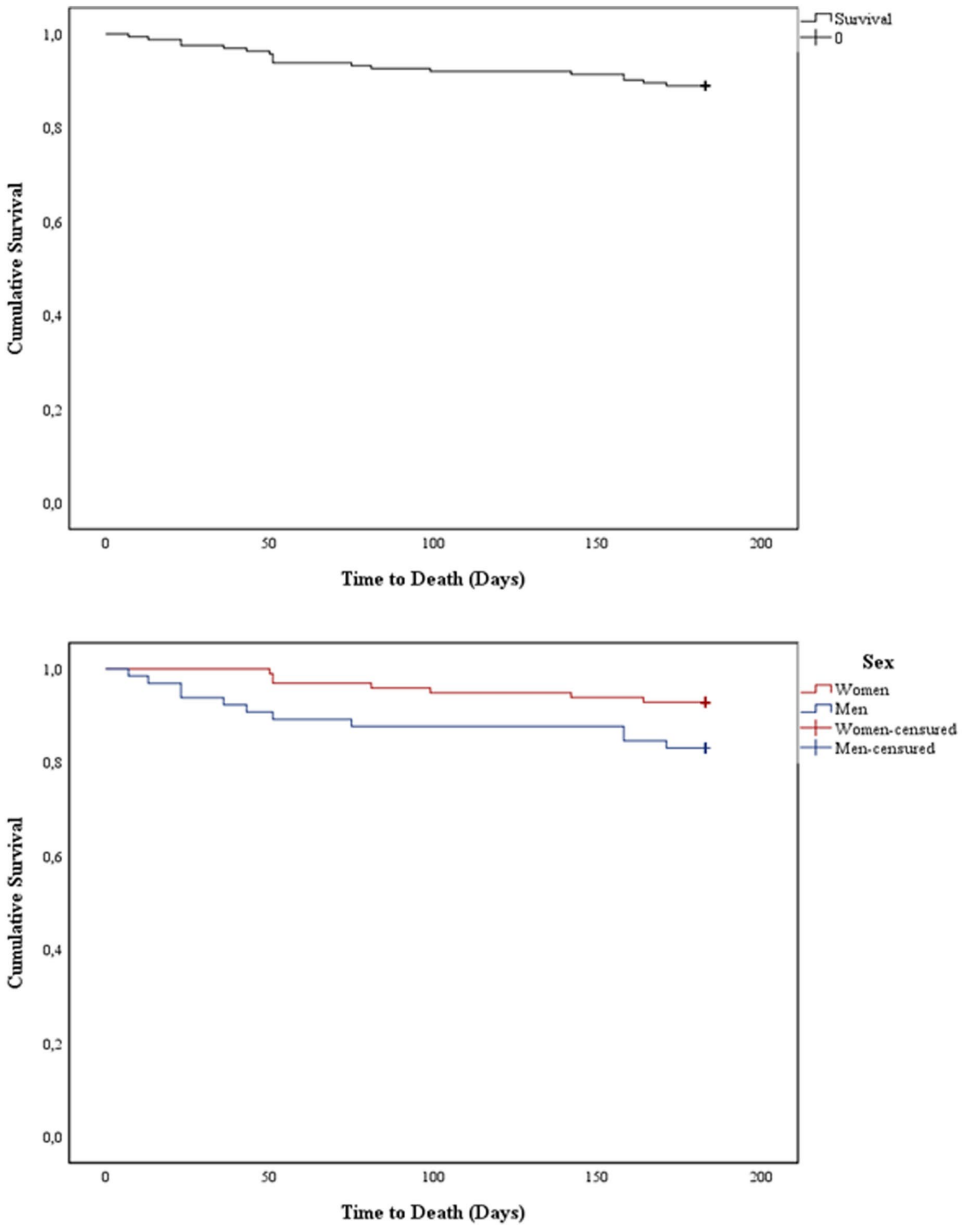
Survival analysis of patients by sex.

Likewise, when evaluating the effect of the surgical delay time on mortality, we found that for each day of delay in addition to the mean of our population (8 days), the risk of instantaneous death increased by 9.7% (HR = 1.097, 95% CI 1.035–1.164, p = 0.002). Survival curve was better for women than men (p = 0.045) (Fig. 2).

### Health-related quality of life

**Figure 3:**
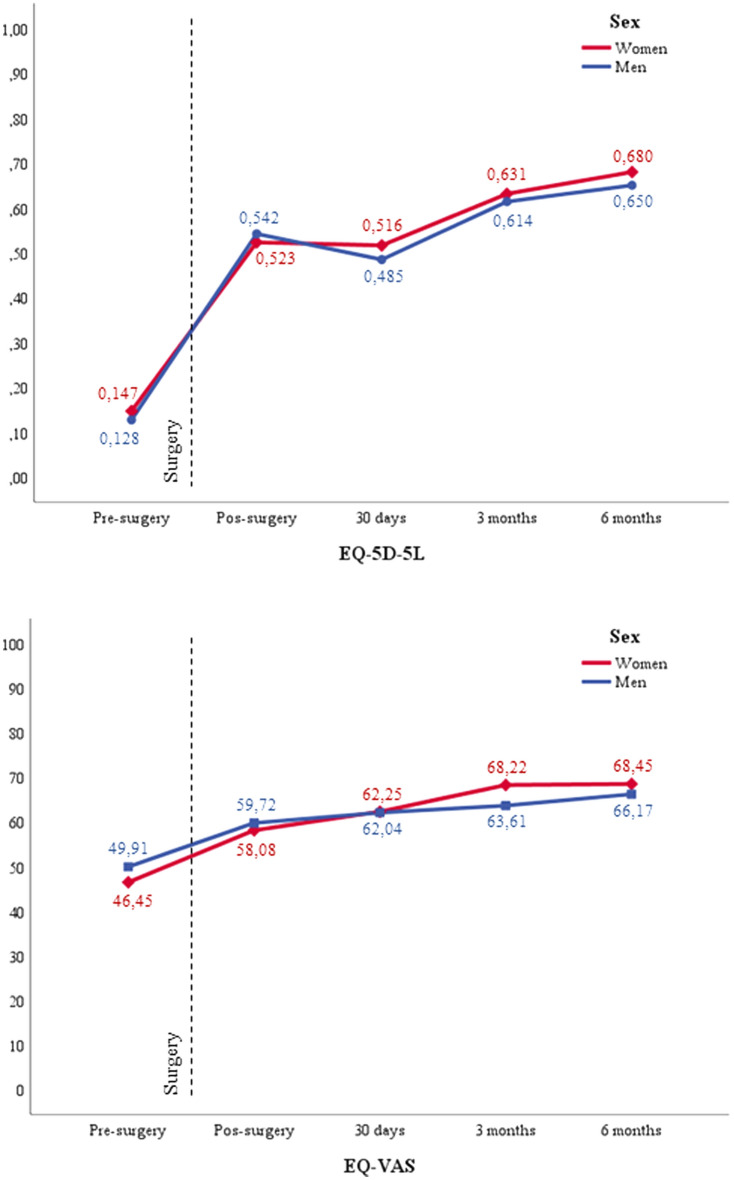
EQ-5D index score and EQ-5D VAS assessment at different follow-up times.

EQ-5D-5L index values increased in comparison with preoperative assessment at 30 days’ follow-up to 0.504 (0.221), at 3 months of follow-up to 0.624 ± (0.199) and at 6 months of follow-up to 0.668 (0.211). EQ-VAS values increased from 62.2 (18.2) at 30 days’ follow-up to 66.5 (18.7) at 3 months of follow-up and 67.6 (21.1) at 6 months of follow-up (Supplementary Table 3). There was an improvement in the evolution by time for both, EQ-5D index score and EQ-5D VAS assessment, p < 0.001; but there were no differences between the sexes (p = 0.429 and p = 0.853, respectively). Figure 3 shows trends in the mean index for EQ-VAS and EQ-5D-5L by sex during 6 months follow-up.

## Discussion

The mean surgical delay from hospital admission was 8.3 days and, from the moment of the fracture to surgery, 11.8 days. This time to surgery exceeds by and large, those recommended by different clinical practice guidelines and studies that have assessed the effect of time on the prognosis of patients with PFF^25–27^. Three days, patients delayed their visit to the hospital. This could be explained by the patient's decision adjournment their visit to the hospital^28,29^, by the geographic location that limits their access to health care, or because they are referred from other hospitals to a reference center for definitive treatment^17,28,30,31^. Once admitted to the hospital, main reason for the surgical delay was administrative such as the authorization of the surgery; second cause was medical issues such as performing additional pre-surgical examinations. In contrast, in other study, stabilizing an existing disease has been the main reason for the delay^32^. Administrative causes of delay to surgery has been associated with a higher risk of complications and mortality at one year when compared with delay due to medical reasons^32^.

These two large groups (medical and administrative factors) related to the delay, correspond to those identified by two reviews that highlight the socioeconomic level, the stabilization of comorbidities and the availability of the operating room as the main reasons for proximal fracture surgery of the proximal femur fracture to 48 h^18,19^. However, the evidence is variable, few studies report the same events or measures them in a homogeneous way, which limits proposing targeted actions to intervene in this problem. In this sense, a study in the United Kingdom that identified access to the operating room as the leading cause of surgical delay reported that implementing an economic incentive to hospitals resulted in additional operating rooms and rethinking the prioritization of surgical shifts^33^.The hospital stay in our study was around 13 days, longer than the reported in Europe^3,12^ with hospitalizations close to 10 days; but they were similar to the results reported in another Colombian study and United Kingdom^3,17,28^. Surgical delay possibly strongly influenced the hospital stay.

Included population has a demographic distribution similar to other studies, predominantly women and adults with a mean age of eighty years old^3,5,11,12,17^. Likewise, with age, the number of previous diseases increases and in consequence the surgical risk, factors that have influenced the time to surgery and mortality in our study. Cardiovascular and endocrine diseases were the main pre-existing pathologies in the patients included in our cohort, in agreement with other studies carried out in various countries^12,14,16,28,34^; this condition probably affects the need for more time for medical optimization before surgery^35^. The mean Charlson comorbidity index of the patients was 4.6, which estimates a survival of less than 50% at 10 years^20^, similar to that reported in other studies^12^. On the other hand, we found an ASA II lower than that described in most hip fracture studies, suggesting differences between studies in the characteristics of the population included^3,12,17,35,36^.

Most patients suffered an intertrochanteric fracture when other studies have shown that subcapital fractures increase progressively with age^37^. The patients were treated with intramedullary osteosynthesis (> 80%) consistent with the type of fracture. The use of the spinal anesthesia was higher than in other cohort studies^3,12,36^ and it could be due to the variability in clinical practice given the lack of consensus to recommend one technique over the other regarding complications^25,37^. In the included patients the most frequent intraoperative complication was hypotension, which is a common side effect of spinal anesthesia and it occurs in 16–33% of cases^38^. In previous studies hypotension has been associated with significant increases of postoperative mortality^25^.

However, mortality at 1 month was next to 3%, comparable with other studies^12^, but lower than that reported in Europe and another Colombian study^3,17,36^. At 6 months of follow-up, the cumulative mortality was 11%, a result included in the range from 7 to 25% reported by other studies^16,17^.

Delay in surgical time have been described as predictors of mortality^26,27,39^. In our study, as most surgeries were delayed, we could not analyze the effects of it on mortality.

When evaluating the HRQoL, a significant improvement with respect to the pre-surgical state was found both in the EQ-5D-5L index and in the VAS at 1 month at 3 and 6 months; these estimates are greater to those described in Spain and Thailand^5,11,12^. Higher HRQoL estimates reported could be explained by the baseline conditions of the patients, such as a lower ASA, do not being institutionalized or have not presenting dementia, compared with the results of other authors^4,6,8^. In agreement with various studies, the greatest change in quality of life occurred in the first 3 months after surgery and thereafter the improvement was not remarkable^4,6,8^.

There were no differences between sexes in age, type of fracture and quality of life during the follow-up period, although the causes of the fracture differed between them, possibly related to the overweight and the presence of osteoporosis, secondary to hormonal suppression in menopause that makes women more susceptible to fractures despite having low-energy trauma^38–40^. Mortality was higher in men than in women and could be explained by the fact that men received more hip replacements and developed more postoperative complications^36,39,41,42^.

Colombia is one of the countries with the longest delays in carrying out a procedure^42,43^. Furthermore, rural predominance of patients, distant from the levels of care, the use of traditional medicine and low levels of schooling can influence the time to consultation with medical services^29^. For the above, an intervention by government entities is essential to modify these notable delays. In addition, other measures should be implemented such as an improvement in infrastructure adapted to the needs of the population, a rapid referral of rural patients, adequate supplies, hospital beds, critical care units, operating rooms, and sufficient human talent in health to provide early, comprehensive, and good quality care for patients with hip fractures.

The HIPCCO is the first prospective study in Colombia to analyze surgical delay factors and their impact on the quality of life of patients with PFF and one of the few prospective cohorts with follow-up of patients with proximal femur fractures carried out in middle-income countries. Being data collection prospective collected, the information bias was avoided. In addition, the quality of life of the patients was evaluated using the validated EQ-5D-5L instrument^3^.

As limitations of our study, it was unicentric, from a region with a low population density. Selection bias could be incurred because were included patients undergoing surgery but not patients who received conservative management for femoral fracture. Like-wise, not registering the quality of life prior to the fracture at the time of admission to the hospital, did not allow evaluating the postsurgical results with the pre-fracture state of the patient. On the other hand, given that there are very limited data on Colombian HRQoL that make it difficult to compare these results at the local level, US utilities have been used in accordance with EuroQol recommendations^44–48^.

Work should be done in middle-income countries such as Colombia, to improve patient access to hospital care and reduce administrative factors that delay hip surgery, which is a modifiable risk factor for mortality and complications in this group of patients. Despite this, results obtained in this cohort reflect low mortality and a level of HRQoL comparable with studies in other latitudes.

## Supplementary Information

Supplementary Information.

## Data Availability

Explaining the reasons for the requirement, data and materials of the study will be available contacting with authors.

## References

[1] Burgers P (2016). Total medical costs of treating femoral neck fracture patients with hemi-or total hip arthroplasty: A cost analysis of a multicenter prospective study. Osteoporos. Int..

[2] Hektoen LF (2016). One-year health and care costs after hip fracture for home-dwelling elderly patients in Norway: Results from the Trondheim Hip Fracture Trial. Scand. J. Public Health.

[3] Ojeda-Thies C (2019). Spanish National Hip Fracture Registry (RNFC): Analysis of its first annual report and international comparison with other established registries. Osteoporos. Int..

[4] Gabbe BJ (2016). Return to work and functional outcomes after major trauma. Ann. Surg..

[5] Ruiz-Romero MV (2020). Influencia de la cirugía precoz de la fractura de cadera en ancianos en la mortalidad, los reingresos, la dependencia y la calidad de vida. Rev Esp Salud Publica.

[6] Brown K, Cameron ID, Keay L, Coxon K, Ivers R (2017). Functioning and health-related quality of life following injury in older people: A systematic review. Inj. Prev..

[7] Alexiou KI, Roushias A, Varitimidis SE, Malizos KN (2018). Quality of life and psychological consequences in elderly patients after a hip fracture: A review. Clin. Interv. Aging.

[8] Peeters CM (2016). Quality of life after hip fracture in the elderly: A systematic literature review. Injury.

[9] Tang VL (2017). Rates of recovery to pre-fracture function in older persons with hip fracture: An observational study. J. Gen. Intern. Med..

[10] Aziziyeh R (2019). The burden of osteoporosis in four Latin American countries: Brazil, Mexico, Colombia, and Argentina. J. Med. Econ..

[11] Amphansap T, Sujarekul P (2018). Quality of life and factors that affect osteoporotic hip fracture patients in Thailand. Osteoporos. Sarcopenia.

[12] Amarilla-Donoso FJ (2020). Quality of life after hip fracture: A 12-month prospective study. PeerJ.

[13] Rivillas-Garcia JC, Gómez-Aristizabal LY, Rengifo-Reina HA, Muñoz-Laverde EP (2017). Envejecimiento poblacional y desigualdades sociales en la mortalidad del adulto mayor en Colombia¿ Por qué abordarlos ahora y dónde comenzar?. Rev Fac Nac Salud Pública.

[14] Pincus D (2018). Reporting and evaluating wait times for urgent hip fracture surgery in Ontario, Canada. CMAJ.

[15] Leicht H (2021). Time to surgery and outcome in the treatment of proximal femoral fractures. Dtsch. Ärztebl. Int..

[16] Morales Ó, Parra JD, Mateus R (2018). Morbimortalidad posterior a fracturas intertrocantéricas de cadera. Efecto del retraso en el tratamiento quirúrgico. Rev. Colomb. Ortop. Traumatol..

[17] Espinosa KA, Gélvez AG, Torres LP, García MF, Peña OR (2018). Pre-operative factors associated with increased mortality in elderly patients with a hip fracture: A cohort study in a developing country. Injury.

[18] Sheehan KJ, Sobolev B, Villán YFV, Guy P (2017). Patient and system factors of time to surgery after hip fracture: A scoping review. BMJ Open.

[19] Xu BY, Yan S, Low LL, Vasanwala FF, Low SG (2019). Predictors of poor functional outcomes and mortality in patients with hip fracture: A systematic review. BMC Musculoskelet. Disord..

[20] Charlson ME, Pompei P, Ales KL, MacKenzie CR (1987). A new method of classifying prognostic comorbidity in longitudinal studies: Development and validation. J. Chronic Dis..

[21] Hocevar, L. A. & Fitzgerald, B. M. American society of anesthesiologists staging. In *StatPearls* (2019). 31747192

[22] EuroQol Research Foundation. EQ-5D-5L User Guide. (2019).

[23] Pickard AS (2019). United States valuation of EQ-5D-5L health states using an international protocol. Value Health.

[24] Von Elm E (2014). The strengthening the reporting of observational studies in epidemiology (STROBE) statement: Guidelines for reporting observational studies. Int. J. Surg..

[25] Griffiths R (2021). Guideline for the management of hip fractures 2020: Guideline by the Association of Anaesthetists. Anaesthesia.

[26] Beaupre LA (2019). The impact of time to surgery after hip fracture on mortality at 30-and 90-days: Does a single benchmark apply to all?. Injury.

[27] Leer-Salvesen S (2019). Does time from fracture to surgery affect mortality and intraoperative medical complications for hip fracture patients? An observational study of 73557 patients reported to the Norwegian Hip Fracture Register. Bone Jt. J..

[28] Paul P, Issac RT (2018). Delay in time from fracture to surgery: A potential risk factor for in-hospital mortality in elderly patients with hip fractures. J. Orthop..

[29] Russo M (2015). Estudio exploratorio del impacto del alfabetismo funcional sobre conductas sanitarias deficientes a nivel poblacional. Rev. Médica Chile.

[30] He W (2020). Admission delay is associated with worse surgical outcomes for elderly hip fracture patients: A retrospective observational study. World J. Emerg. Med..

[31] Butler SA, Salipas A, van Der Rijt A (2019). Comparative study of outcomes for elderly hip fractures presenting directly to a referral hospital versus those transferred from peripheral centres. ANZ J. Surg..

[32] Lizaur-Utrilla A (2019). Reasons for delaying surgery following hip fractures and its impact on one year mortality. Int. Orthop..

[33] Murphy LE, McKenna SM, Shirley D (2015). The best practice tariff and hip fractures: How can Northern Ireland keep up?. The Surgeon.

[34] Barceló M, Torres OH, Mascaró J, Casademont J (2021). Hip fracture and mortality: Study of specific causes of death and risk factors. Arch. Osteoporos..

[35] Declarador N, Ramason R, Tay L, Chan WLW, Kwek EBK (2018). Beyond comanaged inpatient care to community integration: Factors leading to surgical delay in hip fractures and their associated outcomes. J. Orthop. Surg..

[36] Åhman R (2018). Determinants of mortality after hip fracture surgery in Sweden: A registry-based retrospective cohort study. Sci. Rep..

[37] Tanner DA, Kloseck M, Crilly RG, Chesworth B, Gilliland J (2010). Hip fracture types in men and women change differently with age. BMC Geriatr..

[38] Samuelsson B (2009). Gender differences and cognitive aspects on functional outcome after hip fracture—A 2 years’ follow-up of 2,134 patients. Age Ageing.

[39] Sterling RS (2011). Gender and race/ethnicity differences in hip fracture incidence, morbidity, mortality, and function. Clin. Orthop. Relat. Res..

[40] González MÁ, Hernández R, Malagón JM, García A, Manrique J (2021). Perfil epidemiológico de los pacientes adultos mayores de 65 años con fractura de cadera. Estudio de Cohorte Transversal. Rev. Colomb. Ortop. Traumatol..

[41] Negrete-Corona J, Alvarado-Soriano JC, Reyes-Santiago LA (2014). Fractura de cadera como factor de riesgo en la mortalidad en pacientes mayores de 65 años: Estudio de casos y controles. Acta Ortopédica Mex..

[42] Kannegaard PN, van der Mark S, Eiken P, Abrahamsen BO (2010). Excess mortality in men compared with women following a hip fracture. National analysis of comedications, comorbidity and survival. Age Ageing.

[43] Abrahamsen B, Van Staa T, Ariely R, Olson M, Cooper C (2009). Excess mortality following hip fracture: A systematic epidemiological review. Osteoporos. Int..

[44] Bailey HH, Janssen MF, Varela RO, Moreno JA (2021). EQ-5D-5L population norms and health inequality in Colombia. Value Health Reg. Issues.

[45] Rojas-Reyes MX, Gomez-Restrepo C, Rodríguez VA, Dennis-Verano R, Kind P (2017). Calidad de vida relacionada con salud en la población Colombiana: ¿cómo valoran los colombianos su estado de salud?. Rev. Salud Pública.

[46] Santos AM (2018). Calidad de vida evaluada por EQ-5D-3L de los enfermos reumáticos en Colombia. Med. Bogotá.

[47] Cano Gutiérrez C (2016). Perception of health-related quality of life using the EURO-QOL in older adults in Bogotá Colombia. Eur. Geriatr. Med..

[48] Viana Barceló RA, Navarro España JL (2018). Estado de salud de los colombianos: una aplicación del EQ-5D-3L. Arch. Med. Manizales.

