# Supplementary material for: Delayed surgery and health related quality of life in patients with proximal femoral fracture
Source: Sci Rep. 2023 Jul 10;13:11131. doi: 10.1038/s41598-023-33592-3 (PMC10333196; doi:10.1038/s41598-023-33592-3)
Supplement: Supplementary file 1 — Supplementary Information. [file 41598_2023_33592_MOESM1_ESM.docx]

**Supplementary table 1.** Postoperative adverse event

| Variables | Total (n=163)  n (%) | Female (n=98) | Male (n=65)  n (%) | p |
| --- | --- | --- | --- | --- |
| Post-surgical events/complications | 124 (76.1) | 73 (74.5) | 51 (78.5) | 0.349 |
| Anemia | 88 (54.0) | 54 (55.1) | 34 (52.3) | 0.424 |
| Pain | 69 (42.3) | 34 (34.7) | 35 (53.8) | 0.015 |
| Hydroelectrolyte disorder | 34 (20.9) | 21 (21.4) | 13 (20.0) | 0.494 |
| Delirium | 14 (8.6) | 8 (8.2) | 6 (9.2) | 0.512 |
| Respiratory infection | 7 (4.3) | 4 (4.1) | 3 (4.6) | 0.580 |
| Urinary infection | 5 (3.1) | 4 (4.1) | 1 (1.5) | 0.336 |
| Operative site infection | 5 (3.1) | 2 (2.0) | 3 (4.6) | 0.313 |
| Pressure ulcers | 4 (2.5) | 1 (1.0) | 3 (3.6) | 0.175 |
| Deep venous thrombosis | 3 (1.8) | 1 (1.0) | 2 (3.1) | 0.349 |
| Cardiovascular events | 1 (0.6) | 1 (1.0) | 0 (0) | 0.601 |
| Duration of events Median (IQR) | 2.0 (2-4) | 2.0 (2-4) | 3.0 (2-5) | 0.306 |
| Total length of hospital stay  Mean (SD) | 13.5 (10.3) | 11.8 (8.8) | 16 (11.1) | 0.011 |

|  |
| --- |

**Supplementary table 2.** Factors influencing Length of Stay

|  | **β** | **Delay time** |  | **p value** | **Lower limit CI95%** | **Upper limit CI95%** |
| --- | --- | --- | --- | --- | --- | --- |
| **BIVARIANT MODEL** | | | | | | |
| Age | -0.167 | -0.115 | 0.054 | **0.033** | -0.221 | -0.010 |
| Sex | 0.200 | 4.224 | 1.634 | **0.011** | 0.997 | 7.452 |
| Subsidized insurance regime | 0.138 | 2.894 | 1.640 | 0.080 | -.0345 | 6.134 |
| Osteosynthesis material non-availability | 0.060 | 1.691 | 2.226 | 0.448 | -2.705 | 6.088 |
| Request of additional preoperative test | 0.136 | 2.987 | 1.719 | 0.084 | -0.408 | 6.381 |
| Charlson index | 0.098 | 0.541 | 0.432 | 0.213 | -0.313 | 1.395 |
| Operating room scheduling delay | -0.005 | -0.131 | 2.107 | 0.951 | -4.292 | 4.031 |
| Request of additional medical specialties assessment | 0.198 | 4.344 | 1.693 | **0.011** | 1.001 | 7.687 |
| Delay in authorization of surgery | 0.191 | 4.719 | 1.914 | **0.015** | 0.940 | 8.498 |
| Decompensated basal pathology | 0.198 | 5.355 | 2.093 | **0.011** | 1.221 | 9.488 |
| Medical events generated during admission | 0.234 | 7.087 | 2.324 | **0.003** | 2.499 | 11.676 |
| **FINAL MODEL** | | | | | | |
| Delay in authorization of surgery | 0.315 | 3.674 | 0.822 | **0.000** | 2.051 | 5.297 |
| Medical events generated during admission | 0.225 | 3.214 | 1.010 | **0.002** | 1.220 | 5.208 |
| Request of additional medical specialties assessment | 0.230 | 2.369 | 0.730 | **0.001** | 0.928 | 3.810 |
| Decompensated basal pathology | 0.160 | 2.037 | 0.897 | **0.025** | 0.265 | 3.809 |

**Supplementary table 3. EQ-5D index score and EQ-5D VAS assessment at different follow-up times for 145 patients have all values (91 women and 54 men)**

|  | **Preoperative** | | | **Postsurgical** | | | **30 days** | | | **3 months** | | | **6 months** | | | **p** |
| --- | --- | --- | --- | --- | --- | --- | --- | --- | --- | --- | --- | --- | --- | --- | --- | --- |
|  | ♀ | ♂ | Total | ♀ | ♂ | Total | ♀ | ♂ | Total | ♀ | ♂ | Total | ♀ | ♂ | Total |  |
| EQ-5D VAS |  |  |  |  |  |  |  |  |  |  |  |  |  |  |  |  |
| Mean | 46.4 | 49.9 | 47.7 | 58.1 | 59.7 | 58.7 | 62.2 | 62.0 | 62.2 | 68.2 | 63.6 | 66.5 | 68.4 | 66.2 | 67.6 | **<0.001** |
| SD | 23.7 | 18.6 | 21.9 | 16.9 | 14.4 | 15.9 | 18.5 | 18.0 | 18.2 | 18.6 | 18.8 | 18.7 | 21.3 | 20.8 | 21.1 |  |
| EQ-5D index |  |  |  |  |  |  |  |  |  |  |  |  |  |  |  |  |
| Mean | 0.147 | 0.127 | 0.139 | 0.523 | 0.542 | 0.529 | 0.516 | 0.485 | 0.504 | 0.631 | 0.614 | 0.624 | 0.679 | 0.650 | 0.668 | **<0.001** |
| SD | 0.136 | 0.109 | 0.127 | 0.125 | 0.135 | 0.128 | 0.217 | 0.229 | 0.221 | 0.199 | 0.199 | 0.199 | 0.201 | 0.228 | 0.211 |  |
